# The management and outcome of women with post-hydatidiform mole ‘low-risk’ gestational trophoblastic neoplasia, but hCG levels in excess of 100 000 IU l^−1^

**DOI:** 10.1038/sj.bjc.6605529

**Published:** 2010-02-16

**Authors:** S McGrath, D Short, R Harvey, P Schmid, P M Savage, M J Seckl

**Affiliations:** 1 Department Medical Oncology, Charing Cross Hospital Trophoblastic Disease Screening and Treatment Centre, Imperial College NHS Healthcare Trust, Fulham Palace Rd, London W68RF, UK

**Keywords:** GTN, chemotherapy, hCG

## Abstract

**Background:**

Gestational trophoblastic neoplasia (GTN) after a hydatidiform mole is either treated with single- or multi-agent chemotherapy determined by a multifactorial scoring system. Women with human chorionic gonadotrophin (hCG) levels >100 000 IU l^−1^ can remain within the low-risk/single-agent category and usually choose one drug therapy. Here we compare the success and duration of single- *vs* multi-agent chemotherapy in this patient group.

**Methods:**

Between 1980 and 2008, 65 women had a pre-treatment hCG >100 000 IU l^−1^ and were low risk. The initial hCG level, treatment regimens, changes and duration and overall survival were recorded.

**Results:**

Of 37 patients starting low-risk/single-agent treatment, 11 (29.7%) were treated successfully, whereas 26 (70.3%) required additional multi-agent chemotherapy to achieve complete remission (CR). Combination chemotherapy was initially commenced in 28 women, and 2 (7%) required additional drugs for CR. The overall duration of therapy for those commencing and completing single- or multi-agent chemotherapy was 130 and 123 days (*P*=0.78), respectively. The median-treatment duration for patients commencing single-agent but changing to multi-agent chemotherapy was 13 days more than those receiving high-risk treatment alone (136 *vs* 123 days; *P*=0.07). All 3 patients with an initial hCG >400 000 IU l^−1^ and treated with single-agent therapy developed drug resistance. Overall survival for all patients was 100%.

**Conclusion:**

Low-risk post-molar GTN patients with a pre-treatment hCG >100 000 and <400 000 IU l^−1^ can be offered low-risk single-agent therapy, as this will cure 30%, is relatively non-toxic and only prolongs treatment by 2 weeks if a change to combination agents is required. Patients whose hCG is >400 000 IU l^−1^ should receive multi-agent chemotherapy from the outset.

Gestational trophoblastic disease (GTD) is a spectrum of pregnancy-related disorders the commonest of which are the pre-malignant complete (CM) and partial (PM) hydatidiform mole (Sebire and Seckl, 2008). In the United Kingdom, CM and PM affects 1 and 3 per 1000 pregnancies, respectively. They usually present with vaginal bleeding in the first trimester or with abnormal routine ultrasonography at 10–12 weeks gestation (reviewed in Sebire and Seckl, 2008). Initial treatment involves uterine evacuation, which also enables a histopathological diagnosis. In the vast majority of cases, any residual disease resolves spontaneously over time but in 16% of CM and 0.5% of PM, persisting disease can develop (Seckl *et al*, 2000). Fortunately, GTD including CM and PM all produce the pregnancy hormone human chorionic gonadotrophin (hCG). In women where residual CM or PM is dying out, the hCG concentration in serum and/or urine falls. However, a plateaued or rising hCG is an early sign of persisting disease which usually requires chemotherapy, so hCG follow-up is crucial.

In the United Kingdom, all women with GTD are registered for hCG follow-up with one of three centres located in Dundee, Sheffield and London and treated in either of the latter two. Women with persisting disease are only considered for repeat uterine evacuation in the UK when the hCG is <5000 IU l^−1^ and ultrasound imaging suggests that the disease is confined to the uterine cavity. Analysis in our centre has shown that once the hCG exceeds 5000 IU l^−1^, 70% of patients, despite a second evacuation, will require chemotherapy usually in the form of single-agent methotrexate and folinic acid (MTX–FA) (Savage and Seckl, 2005). This has relatively few side effects and is curative in most, whereas a further evacuation carries a small risk of uterine perforation, especially if endometrial invasion is present. Moreover, if the hCG exceeds 100 000 IU l^−1^ and the uterine mass is large, the potential benefit of a second evacuation is very small and the risks of haemorrhage and perforation increase. Similar findings have been reported by others (Pezeshki *et al*, 2004).

Although MTX–FA will help most patients with gestational trophoblastic neoplasia (GTN) following a molar pregnancy, some will require combination chemotherapy most commonly with etoposide, methotrexate and actinomycin D alternating weekly with cyclophosphamide and vincristine (EMA–CO), which is now widely used around the world (Bolis *et al*, 1988; Bagshawe *et al*, 1989; Newlands *et al*, 1991; Kim *et al*, 2004; Turan *et al*, 2006). So how are patients selected for single-agent MTX–FA *vs* multi-agent EMA–CO chemotherapy?

Several factors are important predictors of disease that will become resistant to single-agent chemotherapy, including the size, number and location of metastases, time from the antecedent pregnancy event to treatment, type of antecedent pregnancy, patient age and hCG level (Bagshawe, 1976). These factors form the basis of the International Federation of Gynecology and Obstetrics (FIGO) scoring system, which correlates with the risk of developing disease resistant to single-agent therapy (Kohorn *et al*, 2000). Although the scoring system has been very helpful there are still some areas where it is possible that the information derived for determining the type of therapy could be refined. One such area relates to the level of hCG before chemotherapy. The scoring for hCG concentrations are 0 for <1000 IU l^−1^, 1 for 1000–10 000 IU l^−1^, 2 for 10 000–100 000 IU l^−1^ and 4 for >100 000 IU l^−1^. A total score of 0–6 classifies patients as low-risk, whereas a score >6 identifies patients as high-risk and selects them for multi-agent chemotherapy. Most patients with an hCG of over 100 000 IU l^−1^ score additional points for tumour volume and metastases placing them as >6 but a few women score 6 or less and are therefore technically low-risk. Such patients may have hCG levels that are much higher (e.g., over 500 000 IU l^−1^) and might be expected to have a very small chance of being cured with single-agent therapy.

At our institute, patients with an hCG of >100 000 IU l^−1^ and a FIGO score of ⩽6 have been involved in the decision of whether to treat with MTX–FA or EMA–CO. MTX–FA has little short-term toxicity and no appreciable long-term toxicity (McNeish *et al*, 2002). In contrast, EMA–CO causes alopecia and myelosuppression, brings forward the menopause by 3 years and increases the risk of second malignancies by approximately 1.5 fold (Rustin *et al*, 1996; Bower *et al*, 1998). As a result, many patients select MTX–FA treatment, whereas some choose EMA–CO in the belief that it is more likely to cure them rapidly.

In this study, we have retrospectively compared the outcomes for women with low-risk GTN and a pre-treatment hCG value >100 000 IU l^−1^, treated with either MTX–FA or EMA–CO as their initial therapy. We specifically examine the risk of drug resistance in those commencing single-agent MTX–FA and the total duration of therapy required to induce a sustained remission. We also ask whether there is an hCG level beyond which there is no point in trying MTX–FA therapy in low-risk patients.

## Materials and methods

Between January 1980 and May 2008, 2050 women with post-hydatidiform mole GTN were treated at Charing Cross Hospital. Of these, 174 had an admission hCG value of over 100 000 IU l^−1^. All patients underwent radiological investigation with a Doppler and ultrasound scan of the pelvis and chest X-ray to assess the maximum diameter of the uterine mass and the presence of lung metastases. Patients were stratified according to the Charing Cross system (Bagshawe, 1976) and after 2000, also the FIGO scoring system (Table 1) (Kohorn *et al*, 2000). To simplify the analysis and render it relevant to current practise, all patients scored with the Charing Cross system before 2000 had this value converted to the new FIGO system. Scores totalling 0–6 were classified as low-risk, and those scoring 7 or above were classified as high-risk. Only those patients scoring low-risk were selected for further analysis in this report. These patients were informed of the benefits, toxicity profiles and likelihood of cure for both the low-risk treatment with 8-day MTX–FA (intramuscular MTX 50 mg days 1,3,5 and 7 and oral FA 15 mg days 2, 4, 6 and 8) and high-risk treatment with EMA–CO (intravenous etoposide 100 mg m^−2^ days 1 and 2, MTX 300 mg m^−2^ day 1, actinomycin D 0.5 mg days 1 and 2, cyclophosphamide 600 mg m^−2^ and vincristine 0.8 mg m^−2^ day 8) as previously described (McNeish *et al*, 2002), and were encouraged to participate in the treatment decision process. Serum hCG values were measured twice weekly during treatment and in the presence of three static or two rising hCG values, patients were defined as having drug-resistant disease.

In the case of low-risk patients developing resistance to MTX–FA, treatment was escalated to actinomycin D (0.5 mgs intravenously days 1–5 every 2 weeks) if the hCG level was <100 IU l^−1^ at the point of resistance, or to the EMA–CO regime if the hCG was >100 IU l^−1^ as previously described (McNeish *et al*, 2002). If patients developed side effects from MTX, the FA dose was initially increased, however, if symptoms persisted they were also converted to an alternative regimen as previously described (McNeish *et al*, 2002). For patients developing resistance to EMA–CO, then we have previously added cisplatin by combining with etoposide and alternating weekly with EMA, in which the second day of the EMA is omitted (Newlands *et al*, 2000). More recently, we have developed a less toxic alternative comprising paclitaxel and etoposide alternating two weekly with paclitaxel and cisplatin (TE–TP) (Wang *et al*, 2008).

Treatment was continued for 6–8 weeks after the normalisation of the serum hCG (i.e. <5 IU l^−1^) after which, lifelong hCG monitoring commenced. Relapsed disease was characterised as a rise in serum hCG value ≥6 weeks after normalisation, in the absence of a new pregnancy.

The INSTAT Statistics program was used for the analysis of data using median hCG values and median duration of treatment, and application of the Mann–Whitney test for comparison of these variables.

This retrospective analysis was accepted by our local Institutional Review Board.

## Results

**Figure 1:**
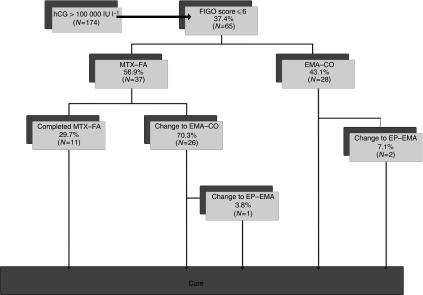
Flow diagram depicting outcomes of patients presenting with human chorionic gonadotrophin (hCG) >100 000 IU l^−1^.

Between January 1980 and May 2008, 65 (37%) of the 174 patients with hCG values >100 000 IU l^−1^ had a FIGO Score of 6 or below (Figure 1). In all, 37 (56.9%) of these 65 patients were commenced on low-risk MTX–FA therapy, with 18 of those scoring 6, 18 scoring 5 and only 1 patient scoring 4, reflecting the fact that an hCG >100 000 warrants 4 points alone. Of the 37 patients commencing MTX–FA, 11 (29.7%) with an initial median hCG reading of 142 473 IU l^−1^ (range 101 510–322 461) were cured without requiring a change of therapy (Figure 1 and Table 2). However, 26 patients (70.3%) with an initial median hCG of 136 287 IU l^−1^ (range 102 199–1 217 592) required a change to EMA–CO. There was no significant difference between these hCG median values (*P*=0.58) as shown in Table 3. The highest hCG value successfully treated by MTX–FA alone was 322 461 IU l^−1^. Three patients had hCG values exceeding this (414 876, 423 563 and 1 217 592), and all required a change to EMA–CO.

All patients who changed from MTX–FA therapy had an hCG >100 IU l^−1^ and so none received actinomycin D (McNeish *et al*, 2002). EMA–CO was given in 26 such patients, 24 because of MTX–FA resistance, 1 because of toxicity and 1 patient relapsed within 6 weeks of completing MTX–FA therapy. In one individual, a further change in treatment from EMA–CO to etoposide and cisplatin alternating weekly with etoposide, methotrexate and actinomycin D (EP–EMA) was made because of an hCG plateau; however, there have not been any other documented relapses in these patients. The overall survival rate for the 65 patients with low-risk scores and hCG >100 000 IU l^−1^ was 100%.

In total, 28 (43.1%) of the 65 patients were commenced on high-risk EMA–CO treatment in the first instance, with 19 having a FIGO score of 6 and 9 with a score of 5. Only two patients required additional chemotherapy to effect a sustained remission (Figure 1). The initial median hCG reading for this group was 239 903 IU l^−1^ (range 102 732–1 843 371). This was significantly higher than the median hCG of those patients commencing and either completing MTX–FA (*P*=0.01) or changing to EMA–CO (*P*=0.002) (Tables 2 and 3).

Comparing those patients that completed MTX–FA, with those who received EMA–CO straight away, there was no significant difference in the total length of treatment (*P*=0.78). The median duration of treatment with MTX–FA alone was 130 days (range 78–180), and the median duration of treatment for EMA–CO was 123 days (range 74–247). There was no significant difference in the median duration of treatment between those patients that completed MTX–FA and those that required a change to EMA–CO that is, 130 days compared with 136 days (*P*=0.07). However, there was a non-significant 13 day increase (*P*=0.07) in treatment duration in those patients commencing MTX–FA but ultimately requiring a change in treatment, compared with those commencing EMA–CO in the first instance (Tables 2 and 3).

## Discussion

Gestational trophoblastic neoplasia is highly responsive to chemotherapy and prognosis is excellent following treatment, especially in low-risk patients (Ngan and Seckl, 2007). In selecting the most appropriate therapy, it is important to minimise toxicity, both in terms of short- and long-term side effects while preserving efficacy.

Several previous studies have examined the link between hCG levels and the likelihood of resistance to MTX. Berkowitz *et al* (1982) noted that resistance to MTX–FA was more common with pre-treatment hCG titres greater than or equal to 50 000 IU l^−1^ but some patients could still be cured. The experience of Bolis *et al* (1987) suggested that drug resistance and relapse rate was related to an hCG value higher than 10 000 IU l^−1^ when using MTX–FA, whereas Gleeson *et al* (1993) demonstrated that 50% of those with a pre-treatment hCG of greater than 650 IU l^−1^, failed primary weekly MTX therapy. Chen *et al* (2004) concluded that single-agent pulse actinomycin D had only modest activity for methotrexate-resistant GTN, with hCG levels being lower in responders (mean 37 *vs* 3634). They postulated that prediction of remission may be more closely associated with hCG levels than with FIGO score alone. However, none of these studies have addressed the outcome for patients with an hCG >100 000 IU l^−1^ who still score in the low-risk category.

The EMA/CO regimen is associated with greater toxicity than low-risk single-agent treatment, including alopecia, myelosuppression, a reduction in the age of menopause and the increased risk of a second malignancy (Rustin *et al*, 1996; Bower *et al*, 1998). As low-risk MTX–FA conveys less toxicity, should patients with low-risk FIGO scores but with high hCG values at least be offered this treatment initially? If resistance does develop, warranting a change in treatment, is the total treatment duration significantly increased, and does there appear to be any difference in the outcome?

In this cohort of 65 patients with hCG values of over 100 000 IU l^−1^ and with low-risk FIGO scores, 37 (56.9%) were commenced on low-risk MTX–FA treatment, the remainder commencing high-risk EMA–CO treatment. Of those patients in the former group, there was a 70.3% chance of having to change to high-risk treatment, and this was mostly as a result of MTX–FA resistance. This compares with 33.2% risk in the McNeish study (McNeish *et al*, 2002), which mainly included lower admission hCG values and therefore lower FIGO score patients. Despite the high rate of treatment resistance, we believe that MTX–FA treatment in low-risk patients is still a viable treatment option as a number of women will be spared the extra toxicity of multi-drug etoposide-based therapy. Many patients are happy to commence initial low-risk therapy, if there is a chance that they can avoid toxicity, as long as it does not compromise their overall outcome.

There was no significant difference in the median hCG values when comparing the group that completed low-risk treatment with those that required a change of treatment. However, once the hCG rose above 400 000 IU l^−1^, which occurred in three patients, all developed MTX–FA resistance. Clearly patient numbers are limited, but these data suggest that if the presentation hCG values exceed 400 000 IU l^−1^, such individuals might best be treated with multi-agent chemotherapy in the first instance, regardless of a low-risk FIGO score. We do not believe that any other study has previously examined the question of whether there is an hCG level beyond which therapy with single-agent MTX–FA may be completely futile in women who still score as low risk.

The remaining 43.1% of patients were commenced on high-risk EMA–CO treatment straight away. It is interesting to note that there was no significant difference in the total duration of treatment when comparing this group with those that completed MTX–FA (123 *vs* 130 days). Hence, it is possible to offer patients the choice between treatments, ensuring that they are fully aware of the side-effect profiles for both regimens and the 70.3% chance of developing MTX–FA resistance. Nevertheless, if they do commence MTX–FA and develop resistance or toxicity warranting a regimen change, there is a non-significant increase in the total duration of treatment. Indeed, if a regimen change is required, length of treatment is likely to increase by just 13 days compared with EMA–CO alone, or by 6 days compared with MTX–FA alone. As the entire treatment duration can vary from 74 to 247 days, a potential increase of 13 days by commencing MTX–FA rather than EMA–CO is probably not of great concern to patients particularly when this is balanced against the toxicity profiles of the two regimens.

There is increasing interest in using pulsed or 5-day actinomycin D rather than MTX–FA as the initial therapy for low-risk treatment, as this single-agent alternative may produce a slightly improved chance of inducing remission (Gilani *et al*, 2005; Osborne *et al*, 2008; Yarandi *et al*, 2008). It is also thought that actinomycin D may result in a shorter duration of treatment. However, our results suggest that the latter treatment is unlikely to produce significantly faster cures, as the much more potent EMA–CO regimen only reduced treatment duration by an average of 1 week compared with MTX–FA. As in our experience, actinomycin D is more toxic than MTX–FA (McNeish *et al*, 2002), we continue to use the latter as initial therapy for low-risk patients.

Reassuringly, there did not appear to be any difference in the outcome between those completing MTX–FA, requiring a regimen change and commencing combination chemotherapy, in terms of overall survival. This study had a 100% overall survival rate; however, as some patients completed treatment as recently as August 2008, we did not have long-term survival data available on all patients.

In summary, our data suggests that it is reasonable to commence the less-toxic MTX–FA therapy in women presenting with low-risk post-molar GTN and an hCG >100 000 and <400 000 IU l^−1^. Moreover, treating patients until methotrexate resistance is evident and then changing to multi-agent chemotherapy, only prolongs treatment by an average of 2 weeks, and does not compromise the long-term survival of patients. However, in those with an hCG >400 000 IU l^−1^, MTX–FA does not appear to work and these individuals will likely do better by commencing therapy with EMA–CO.

## Figures and Tables

**Table 1:** FIGO scoring system for gestational trophoblastic disease

	**FIGO scoring system**			
**Variable**	**0**	**1**	**2**	**4**
Age (years)	<40	⩾40	—	—
Antecedent pregnancy (AP)	Mole	Abortion or unknown	Term	—
Interval, that is end of AP to start of treatment (months)	<4	4–7	7–12	>12
Human chorionic gonadotrophin (hCG) (IU l^−1^)	<1000	1000–10 000	10 000–100 000	>100 000
Largest mass (cm)	<3	3–5	>5	—
Site of metastases	Nil or lungs	Spleen, kidney	GI tract	Brain, liver
No. of metastases	0	1–4	5–8	>8
Previous chemotherapy	—	—	Single agent	>1 drug

**Table 2:** Data comparisons between the three treatment groups in patients presenting with human chorionic gonadotrophin (hCG) >100 000 IU l^−1^ and FIGO score ⩽6

	**MTX–FA**	**MTX–FA+EMA–CO**	**EMA–CO**
Number of patients	11	26	28
Median initial hCG (IU l^−1^)	142 473	136 287	239 903
Range	101 510–322 461	102 199–1 217 592	102 732–1 843 371
Median duration of treatment (days)	130	136	123
Range	78–180	102–181	74–247

Abbreviations: CO=cyclophosphamide+vincristine; EMA=etoposide+methotrexate+actinomycin D; FA=folinic acid; MTX=methotrexate.

**Table 3:** Statistical comparisons between the three treatment groups in patients presenting with human chorionic gonadotrophin (hCG) >100 000 IU l^−1^ and FIGO score ⩽6 (*P*-values obtained using the Mann–Whitney test)

**Regime**	**Median initial hCG (IU l^−1^)**	**Median duration of treatment (days)**
MTX–FA *vs* MTX–FA+EMA–CO	*P*=0.58	*P*=0.07
MTX–FA *vs* EMA–CO	*P*=0.01	*P*=0.78
MTX–FA+EMA–CO *vs* EMA–CO	*P*=0.002	*P*=0.07

Abbreviations: CO=cyclophosphamide+vincristine; EMA=etoposide+methotrexate+actinomycin D; FA=folinic acid; MTX=methotrexate.
